# *He Who Seeks Finds* (Bodily Signals): German Validation of the Interoceptive Attention Scale (IATS) and its Relationship with Subclinical Psychopathology

**DOI:** 10.1080/00223891.2024.2316236

**Published:** 2024-03-13

**Authors:** Markus R. Tünte, Tara M. Petzke, Sebastian Brand, Jennifer Murphy, Michael Witthöft, Stefanie Hoehl, Mathias Weymar, Carlos Ventura-Bort

**Affiliations:** 1 Department of Developmental and Educational Psychology, Faculty of Psychology, University of Vienna, Austria; 2 Vienna Doctoral School Cognition, Behavior and Neuroscience, University of Vienna, Vienna, Austria; 3 Department of Clinical Psychology, Psychotherapy, and Experimental Psychopathology, Johannes Gutenberg-University Mainz, Mainz, Germany; 4 Department of Psychology, Royal Holloway University of london, London, UK; 5 Department of Biological Psychology and Affective Science, Faculty of Human Sciences, University of Potsdam, Potsdam, Germany; 6 Faculty of Health Sciences Brandenburg, University of Potsdam, Potsdam, Germany

## Abstract

Alterations in interoception have been linked to psychopathology. Recent findings suggest that both the attention to and the accuracy of, interoceptive perceptions may be oppositely related to subclinical symptomatology. Thus, providing well-validated tools that tap into these interoceptive processes is crucial for understanding the relation between interoceptive processing and subclinical psychopathology. In the current study (*N* = 642), we aimed to (1) validate the German version of the Interoceptive Attention Scale (IATS; Gabriele et al., 2022), and (2) test the differential association of self-reported interoceptive attention and accuracy with subclinical symptomatology, including alexithymia, depressive, and anxious symptomatology. We observed that a one-factor solution is a well-fitting model for the IATS. Further, the IATS showed good internal consistency, convergent, and divergent validity, but poor test-retest reliability. Self-reported interoceptive attention and accuracy were unrelated to each other. However, IATS scores were positively related to all measures of psychopathology (except depressive symptomatology), whereas self-reported interoceptive accuracy scores showed negative or nonsignificant relations with these. Our data suggest that the IATS is a good instrument to measure self-report interoceptive attention in the German population. Further, we highlight the need to distinguish between constructs of interoception to better understand the relation between interoception and psychopathology.

“Listen to your body!”—just about every wellness and lifestyle influencer has used this sentence at least once when talking about personal wellbeing or making the right decision. The increased popularity of body-focused attention as a tool for improved mental and physical wellbeing mirrors the surge of interest that the scientific community has experienced in the last years on the conscious processing of internal bodily signals, also referred to as interoception (Khalsa et al., 2018). The growing body of research on interoception is in part influenced by recent findings emphasizing its role in different psychological processes (e.g., Dunn et al., 2010; Garfinkel et al., 2014) as well as clinical outcomes. For instance, deficits in interoceptive processing have been related to different psychopathological disorders including autism (Garfinkel et al., 2016; Quadt et al., 2021), drug addiction (Paulus & Stewart, 2014), eating (Jenkinson et al., 2018) and panic disorders (Ehlers, 1993), as well as (sub) clinical symptomatology such as (persistent) somatic symptoms (Schulz et al., 2020; Van den Bergh et al., 2017; Witthöft et al., 2020) and alexithymia (Brewer et al., 2021; Herbert et al., 2011; Murphy et al., 2018). Along with recent findings on the importance of interoception, new proposals for conceptualizing and operationalizing interoception have emerged.

One of the most influential models of interoceptive processing conceptualizes interoception in three different dimensions: interoceptive accuracy, sensibility, and awareness (Critchley & Garfinkel, 2017; Garfinkel et al., 2015). Interoceptive accuracy refers to the behavioral precision in detecting internal bodily sensations (Critchley & Garfinkel, 2017), while interoceptive sensibility reflects beliefs about the perception of interoceptive signals. Last, interoceptive awareness is the metacognitive awareness of interoceptive accuracy (Critchley & Garfinkel, 2017).

The distinction between abilities, beliefs, and their interplay served as a starting point to clarify the differential role of interoception in a wide variety of psychological aspects (e.g., Critchley & Garfinkel, 2017). Recent proposals suggest disentangling not only between the *methods* used but also between the overarching *constructs* of interoception (e.g., Murphy et al., 2019; Suksasilp & Garfinkel, 2022). One of these proposals is the 2 × 2 factorial model of interoceptive abilities (Murphy et al., 2019). The model differentiates between measures that assess self-reported vs. behavioral aspects of interoception as well as between the constructs of interoceptive accuracy and attention (Murphy et al., 2019). Interoceptive accuracy is defined as correctly perceiving the true state of one’s body while interoceptive attention is understood as the degree to which a person attends to or focusses on bodily stimuli.

Classical interoceptive tasks such as the Heartbeat Counting Task (Schandry, 1981) or Whitehead Heartbeat Detection Task (e.g., Kleckner et al., 2015) are suggested as behavioral measures of interoceptive accuracy. Tasks assessing the extent to which interoceptive signals are attended, such experience-sampling methods (Csikszentmihalyi & Larson, 2014), have been proposed to tap onto behavioral interoceptive attention. Regarding self-reported interoceptive accuracy, Murphy and colleagues have recently developed the Interoceptive Accuracy Scale (IAS, Murphy et al., 2020; see for German validation Brand et al., 2023) that evaluates the beliefs about one’s ability to accurately perceive interoceptive signals (e.g., item 1: *I can always accurately perceive when my heart is beating fast*). Among the existing self-report measures of interoception, the Body Awareness Scale of the Body Perception Questionnaire (BPQ; Porges, 1993; Cabrera et al., 2018) has been postulated as a measure of self-reported interoceptive attention. Similarly, subscales of the Multidimensional Assessment of Interoceptive Awareness Version-2 partly assess this construct (Ventura-Bort et al., 2021). However, these scales were not initially conceived to specifically measure this construct. Furthermore, their construct validity may be considerably influenced by participants’ interpretation of the scale (Gabriele et al., 2022), limiting their psychometric appropriateness to measure interoceptive attention (Campos et al., 2021; Gabriele et al., 2022; Desmedt et al., 2022; Suksasilp & Garfinkel, 2022). To overcome these challenges, Gabriele et al. (2022) recently developed the Interoceptive Attention Scale (IATS) to specifically measure beliefs concerning one’s attention to a broad range of interoceptive signals (e.g., item 1. *most of the time my attention is focused on whether my heart is beating fast*).

In line with the 2 × 2 model of interoception, recent empirical evidence supports the distinction between self-reported attention and accuracy (Campos et al., 2021; Gabriele et al., 2022; Murphy et al., 2020; Murphy et al., 2019; Trevisan et al., 2021). Importantly, these two subfacets of interoception seem to show differential associations with subclinical symptomatology. One well-studied case is alexithymia (Brewer et al., 2021), which is apparently related to lower levels of interoceptive accuracy (Herbert et al., 2011; Murphy et al., 2020), but higher levels of self-reported interoceptive attention (Gabriele et al., 2022; Trevisan et al., 2021).

Similarly, the distinction between interoceptive accuracy and attention has been emphasized in other clinical conditions. Individuals at risk for anxiety or depression may show difficulties in accurately processing their bodily signals and generating appropriate adaptive responses to the environment (Barrett et al., 2016, Paulus & Stein, 2006, 2010, but also Adams et al., 2022). As a result, harmless signals (e.g., heartbeats) might be processed as threats. These inaccurate perceptions may lead to hypervigilant tendencies toward interoceptive signals (Paulus & Stein, 2010), which in turn may provoke perceiving symptoms (e.g., general alert) typically associated with these disorders (Barrett et al., 2016).

To sum up, growing evidence supports a distinction between interoceptive attention and accuracy, especially in the self-report domain. Further, both constructs may differentially impact psychopathology-related outcomes. Therefore, providing validated instruments that specifically tap into and differentiate between interoceptive accuracy and attention is crucial for advancing the understanding of interoceptive processing and its relation to psychopathology. Thus, the goal of the current study was twofold:

1. Validating the German version of the Interoceptive Attention Scale (IATS) and
2. Testing the convergent and discriminant validity of self-reported interoceptive accuracy and attention alongside other self-report and behavioral interoceptive indexes as well as considering their relationship with self-reported clinical psychopathology.

Although in the original English version of the IATS, three components emerged, the questionnaire was *a priori* conceived as a one-factor scale (Gabriele et al., 2022). We thus initially expected to find a one-factor structure with good internal consistency and reliability. In line with prior studies (Gabriele et al., 2022; Murphy et al., 2020), we further expected that the IATS would show convergent validity with attention-related, self-report measures of interoception, but discriminant validity with other self-report and behavioral measures of interoceptive accuracy (Gabriele et al., 2022). We also predicted that self-report interoceptive attention and accuracy scores would show opposing relationships with self-report measures of subclinical psychopathology: interoceptive accuracy would show, if any, negative relations to subclinical psychopathology, whereas IATS scores would be positively related to alexithymia, depression, anxiety, and somatic symptoms. Finally, given that self-reported interoception may vary as a function of age and gender (Murphy et al., 2018; Prentice & Murphy, 2022), we also explored whether IATS scores were modulated by these demographic variables.

## Methods

### Sample size and recruitment

Data were collected from two different sources: the University of Vienna (Sample 1, 2), and the University of Potsdam (Sample 3; see Table 1).

### Transparency and openness

Data collection for all studies and samples reported in this project was preregistered (https://aspredicted.org/K2L_8TW). Due to the multicenter approach, which was not known during creation of the preregistration, and the reviewer feedback for the first submission, some changes have been made to the preregistered analysis plan. The main deviation is that we used the largest sample for confirmatory factor analysis (sample 1 in the manuscript, sample 6 in the preregistration). Further, the present manuscript only reports data on those studies mentioned in the preregistration that used the IATS, while the validation of the IAS was published separately (Brand et al., 2023). Data is available *via* https://osf.io/etg73/?view_only=1cebc9b8754c42f2afa793df85a03bb0. Participants who did not report a high proficiency of German, responded too fast or slow in relation to the median of the overall sample (SoSciSurvey variables *DEG_TIME >*100 or *TIME_RSI >*2; Leiner, 2019a) and/or whose age was below 18 or above 70, in line with our preregistered criteria, were excluded. All demographic information is listed in Table 1.

### Sample 1

A total of 400 German-speaking participants took part in this online study investigating body perception. Participants were recruited *via* the online platform prolific (https://www.prolific.co/) and were compensated with approximately 3.50 €. The questionnaires were displayed *via* SoSciSurvey (Leiner, 2019b). Ethical approval was granted by the Ethics Committee of the University of Vienna (reference number: 00705). Following our preregistered criteria, 12 participants were excluded.

### Sample 2

A total of 80 students from the University of Vienna took part in this study investigating automatic imitation and interoception. Participants were interested volunteers or received course credits. Two participants were excluded due to technical issues and one participant due to missing questionnaire data. Further, for the heartbeat detection tasks two participants were excluded due to artifacts in the ECG. Ethical approval was granted by the Ethics Committee of the University of Vienna (reference number: 00706).

### Sample 3

A total of 256 students from the University of Potsdam took part in the study. Participants were recruited *via* Sona Systems (https://www.sona-systems.com/) and were compensated with course credits. Two participants were excluded because of insufficient German proficiency. A total of 59 participants repeated the online study a second time (i.e., to evaluate test-retest reliability). If the survey was incomplete (*N* = 48) or participants fulfilled any of the abovementioned exclusion criteria (*N* = 24), data were not considered for analysis (Leiner, 2019a). The final sample consisted of 254 participants. The project consisted of two parts, an online part followed by voluntary participation lab assessments. A total of 28 participants underwent the online and laboratory session in which the behavioral interoceptive tasks were administered. Ethical approval was granted by the Ethics Committee of the University of Potsdam (Nr: 8/2020).

Similar to the original validation study (Gabriele et al., 2022), Samples 1 and 3 were used to investigate how the interpretation of questionnaires could moderate the interrelation between them. After filling out the IATS, IAS, and body awareness scale of the BPQ-SF, participants were asked for their interpretation of the questionnaires. Data from sample 2 was used to investigate the relationship of the IATS to behavioral measures of interoception.

## Materials

### Questionnaires

#### Interoceptive attention scale (IATS)

Within the 2 × 2 factorial model of interoception (Murphy et al., 2019), the IATS was designed to evaluate self-reported attention to interoceptive cues such as hunger or breathing (Gabriele et al., 2022). The questionnaire consists of 21 items on a 5-point Likert scale, ranging from (1) *disagree strongly* to (5) *strongly agree*, with higher scores implying greater interoceptive attention. The IATS was designed to match the interoceptive sensations described in a related questionnaire measuring interoceptive accuracy. The original version of the IATS was initially translated into German (scientists with extensive knowledge on the topic did the translation). Subsequently, a back-translation was performed by a professional interpreter or a native speaker. The back-translation was revised by the original authors of the IATS.

#### Interoceptive accuracy scale (IAS)

The IAS was created to measure interoceptive accuracy within the 2 × 2 factorial model of interoception (Murphy et al., 2020). The questionnaire consists of 21 items which are rated on a 5-point Likert scale (from *strongly disagree* to *strongly agree)*. In the current samples, the IAS showed good internal consistency (Sample 1, *ω* = .88; Sample 2, *ω* = .81; Sample 3: *ω* = .85).

#### Body perception questionnaire-short form (BPQ-SF)

The BPQ-SF encompasses 46 items scored on a 5-point Likert-scale (ranging from [1] *never* to [5] *always*) grouped in three subscales that measure two distinct constructs. The Body Awareness subscale (26 items) quantifies the proportion of time a person reports being aware of sensations in their body. Recent studies have interpreted it as a potential measure of interoceptive attention (Murphy et al., 2020; Campos et al., 2021). The supra- (15 items) and subdiaphragmatic (6 items) reactivity subscales, assess the subjectively perceived autonomic nervous system reactivity regarding difficulties in the coordination of bodily functions and symptoms of stress and autonomic dysregulation. In the current study, we attempted to use the German version of the BPQ-SF that is available on the official webpage. Awkwardly translated items were rephrased, using a similar translation procedure to the one of the IATS. The BPQ-SF showed good internal consistency (Sample 1: body awareness: *ω* = .95; supradiaphragmatic reactivity: *ω* = .91; subdiaphragmatic reactivity: *ω* = .89; Sample 3: body awareness: *ω* = 90; supradiaphragmatic reactivity: *ω* = .84; subdiaphragmatic reactivity: *ω* = .81).

#### Multidimensional assessment of interoceptive awareness version-2 (MAIA-2)

The MAIA-2 (Eggart et al., 2021; Mehling et al., 2018) focuses on the evaluation of multiple dimensions of interoception throughout its 37 items divided into 8 subscales. The 8 subscales are: Noticing, Not-Distracting, Not-, Attention Regulation, Emotional Awareness, Self-Regulation, Body Listening, and Trust. Each item is rated on a 6-point Likert-scale, ranging from *never* (0) to *always* (5). Overall, the subscales of the MAIA-2 showed acceptable to good internal consistency: *ω*_*Notice*_ = .72, *ω*_*NonDistracting*_ = .85, *ω*_*NotWorrying*_ = .8, *ω*_*AttentionRegulation*_ = .83, *ω*_*EmotionalAwareness*_ = .82, *ω*_*SelfRegulation*_ = .79, *ω*_*BodyListening*_ = .84 and *ω*_*Trusting*_ = .86

#### Interoceptive confusion questionnaire (ICQ)

The ICQ (Brewer et al., 2016) consists of 20 items rated on a a 5-point Likert scale from *does not describe me* (1) to *describes me very well* (5). It is a measure of beliefs regarding interoceptive accuracy. High scores indicate difficulties in interpreting one’s non-affective physiological states. We found acceptable internal consistency (*ω* = .75).

#### Toronto alexithymia scale 20 (TAS-20)

The German version (Bach et al., 1996) of the Toronto Alexithymia Scale (TAS-20; Bagby et al., 1994) was used to measure alexithymia. The TAS-20 consists of 20 items rated on a 5-point Likert scale (ranging from [1] *strongly disagree* to [5] *strongly agree*). In the current study, TAS-20 showed good internal consistency (Sample 1: *ω* = .89, Sample 3: *ω* = .83).

#### State-trait anxiety inventory, trait inventory (STAI-T)

The STAI-T (Spielberger, 1983) measures trait anxiety. This subscale consists of 20 items rated on a 4-point Likert-scale (from (1) *almost never* to (4) *almost always*). In the current sample, STAI-T showed good internal consistency (Sample 3, *ω* = .93).

#### Anxiety sensitivity inventory (ASI-3)

The anxiety sensitivity inventory-3 (ASI-3; Kemper & Finnern, 2011; Taylor et al., 2007) measures anxiety sensitivity, a construct referring to a person’s fear of their physiological anxiety-related arousal response. The ASI-3 consists of 18 items rated on a 5-point Likert-scale (1 = *very little*; 5 = *very much*) grouped into three subscales: somatic concerns, social concerns, and cognitive concerns. In the current sample, the ASI-3 showed good internal consistency (Sample 3; somatic concerns: *ω* = .85; social concerns: *ω* = 76; cognitive concerns: *ω* = .75, total scores: *ω* = .88).

#### Beck depression inventory (BDI-II)

The Beck Depression Inventory (BDI-II; Beck et al., 1996; Kühner et al., 2007) measures the severity of depressive symptoms. It consists of 21 groups of statements assessing the presence of psychological and bodily symptoms of major depression. Statements are assigned point values (ranging from 0 to 3) reflecting the gravity of depressive symptoms in the probed domain during the last week. In the current sample, the BDI-II showed good internal consistency (Sample 3, *ω* = .94).

### Experimental tasks

Participants from Samples 2 (*N* = 80) and 3 (*N* = 28) performed a heartbeat-counting task (HCT; Schandry, 1981). In each trial of the HCT, participants were instructed to silently count their heartbeats without actively touching any body part in which pulsations could be felt. Participants were further encouraged not to guess their heartbeats, but to count only felt heartbeats (Desmedt et al., 2022). To ensure that the interoceptive accuracy scores extracted from the HCT did not reflect any counting strategy (e.g., estimation of the heartbeats based on the time passed) a time estimation control task was administered (second counting task, SCT; Desmedt et al., 2020; Murphy et al., 2018). In the SCT, participants are instructed to count the number of seconds that have passed in a specific time interval. Each trial began and ended with an acoustic signal. After hearing the tone signaling the end of a trial, participants were instructed to type in the number of heartbeats felt or seconds counted, as well as their confidence in their response. Participants in Sample 2 completed 3 trials in each task (HCT: 35, 45, 105; SCT: 28, 48, 103s), while participants in Sample 3 completed 4 trials in each task (HCT: 25, 35, 45, 100s; SCT: 28, 38, 48, 103s). Trials were presented randomly within blocks and trial length was counterbalanced across participants.

Further, participants from Sample 2 (*N* = 80) also performed a heartbeat detection task (HDT; e.g., Kleckner et al., 2015). The HDT consisted of a total of 40 trials (20 synchronous and 20 asynchronous trials). In each trial, participants were presented with a series of 10 sounds that were presented either synchronously (250 ms after the R-peak) or asynchronously (550 ms after the R-peak) with their heartbeat. Participants had to indicate after each trial whether the presented sounds were synchronous with their heartbeat by pressing a key. Trials were presented in randomized order with intertrial intervals of (3s).

During the experiment, participants’ electrocardiography (ECG) was continuously measured (Sample 2: ADInstruments PowerLab 4/35 and Bio Amp FE 231; Sample 3: MP-160, BIOPAC systems, Goleta, CA). Trials were inspected for artifacts using automatic peak detection and custom dashboards and Matlab scripts. We further collected other relevant information such as Body Mass Index (BMI) and knowledge of one’s resting heart rate.

#### Heartbeat counting task scoring

Data from Samples 2 and 3 were collapsed to examine the relation between questionnaires and performance on the HCT. From the HCT Interoceptive accuracy (IAcc), interoceptive sensibility and interoceptive awareness were extracted (e.g., Garfinkel et al., 2015). IAcc was derived from the counted heartbeats compared to the objectively measured heartbeats and calculated for each participant and trial (*n* is defined as the number of heartbeats counted or measured).:

$$\;Iacc\;=1-\left(\frac{\left|n_{measured\;}-n_{counted\;}\right|}{n_{measured\;}}\right)*100$$

Interoceptive sensibility (or self-reported interoceptive accuracy) scores were derived from the confidence rating about the counted heartbeats (from 0% to 100%). Interoceptive awareness was defined as the absolute difference between the IAcc score and sensibility score in each trial. To ensure normalization of the data, HCT accuracy scores were log-transformed and averaged across trials and for each task and participant, separately.

#### Heartbeat detection task scoring

Initially we intended to compute percent correct responses as an index of interoceptive accuracy on the HDT. However, for the analysis reported here we opted to use a signal detection theory approach using d-prime.

### Statistical analysis

Analyses were conducted using SPSS (version 27, IBM Corp, 2017), and R (R Core Team, 2013). Within R, we used the packages tidyverse, psych, lavaan, lme4, cocor, and foreign (R Core Team, 2020; Bates et al., 2015; Diedenhofen & Musch, 2015; Revelle, 2020; Rosseel, 2012; Wickham et al., 2019).

### Analysis of the structure of the questionnaire

To examine the structure of the IATS, we first conducted a parallel analysis on Sample 1. We used the output of the parallel analysis to determine the factor structure of the questionnaire (e.g., one-, two- or three-factors). Subsequently, we used confirmatory factor analysis (CFA) to fit the data to the factor structure. CFAs were conducted on Sample 3 and Sample 1 and 3 combined. We used a WLSMV estimator with theta parameterization because of the ordinal nature of the data.

### Internal consistency and test-retest reliability

We calculated internal consistency scores for all questionnaires using McDonald’s Omega. Test-retest reliability was only performed in a subset of participants from Sample 3. After completion of the online Session 1, all participants could freely sign up for the retest (online Session 2). Thus, no time limit was imposed between the initial and retest sessions (see supplement D). However, we restricted the analysis to those participants who performed the retests 200 days or less after the initial session (*N*_participants_ = 57; *M*_*d*ays_ = 61, *SD*_days_ = 49). Test-retest reliability was examined using Pearson’s and Spearman’s correlation indexes and intraclass correlation coefficient (ICC). Given that the days passed between Session 1 and Session 2 varied substantially across participants, we tested whether the test-retest scores were modulated by the time lag between sessions, using multiple regressions with scores of the first session as the dependent variable, and scores of the second session as well as the lag time between sessions (i.e., days passed from first to second session) and the interaction between both as predictors.

### Convergent and discriminant validity and relation to psychopathology questionnaires

The relationship between the IATS, self-report and behavioral measures of interoception as well as psychopathology-related questionnaires was examined using Pearson’s correlations. Lastly, to examine the differential association between self-reported interoceptive attention and accuracy scores and other self-report measures of interoception as well as psychopathology-related questionnaires, we compared the size of the correlations between the IATS and IAS and other criterion variables.

## Results

### Demographic data

See Table 1 for demographic information for each of the samples. To investigate whether age or gender had a relationship with the IATS scores we combined all samples (*N* = 642). Overall, we find that age was negatively correlated with IATS scores (*r* = −0.28, *p* < .001). Furthermore, without accounting for the non-binary individuals, females scored higher than males on the IATS, *t*(634) = 316, *p* < .001, *d* = 0.27.

### Confirmatory analysis of the structure of the questionnaire in two samples

Parallel analysis revealed that only one factor showed an adjusted eigenvalue larger than 1 (Sample 1, eigenvalue = 6.26). In light of the output of the parallel analysis and the eigenvalue criterion, we fit a one-factor model, using confirmatory factor analysis. Results of the fit suggested that the one-factor solution was adequate (see Table 2).

### Internal consistency and test-retest reliability

The IATS showed good internal consistency in Sample 1 (*ω* = .92), and Sample 3 (*ω* = .85). Test-retest reliability appeared to be rather poor for the IATS (Pearson: *r* = .55, Spearman: *ρ* = .57, ICC = .56). Given the low test-retest reliability, exploratory analysis comparing the IATS test-retest socres to those from other interoception questionnaires were performed. We observed that the test-retest reliability of the IATS did not differ from the IAS (Pearson: *r* = .68, Spearman: *ρ* = .56, ICC = .54; *Z* = −1.12, *p* = .26), nor did the body awareness scale differ from the BPQ-SF (Pearson: *r* = .46, Spearman: *ρ* = .46, ICC = .45; *Z* = 0.69, *p* = 0.49). However, it was significantly lower than the supradiaphragmatic (Pearson: *r* = .72, Spearman: *ρ* = .72, ICC = .78; *Z* = −2.41, *p* = .01) and subdiaphragmatic scales of the BPQ-SF (Pearson: *r* = .77, Spearman: *ρ* = .71, ICC = .78; *Z* = −2.24, *p* = .025).

Regression analysis to control for the time lag between Session 1 and 2 showed that scores on Session 2 predicted values from Session 1 (*b* = 0.73, *t*(55) = 3.76, *p* < .001), but neither days passed (*b* = 0.08, *t*(55) = 0.74, *p* = .46), nor its interaction with scores from Session 2 predicted scores from Session 1 (*b* = −0.00, *t*(55) = −0.8, *p* = .43), indicating that the stability of the scores was independent of the time passed between sessions.

### Convergent validity with interoception-related and other questionnaires and version similarities

See Table 3 for correlations between the IATS and other interoception-related and psychopathology-related questionnaires.

**Figure 1:**
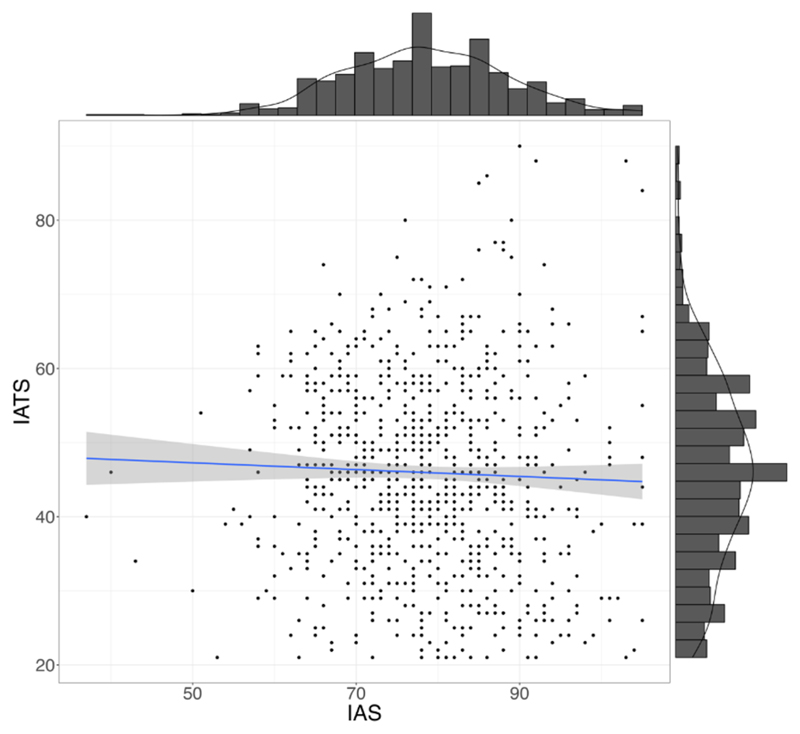
Scatterplot and linear regression of the relationship between IAS and IATS across samples (*N* = 642, *r* = −0.03, *p* = .48). *Note*. IATS = interoceptive attention scale; IAS = interoceptive accuracy scale

To investigate whether self-reported interoceptive accuracy and attention were related, we combined samples 1 and 3 and correlated IAS and IATS scores for all participants that filled out both questionnaires (*N* = 642). Overall, we did not find a significant correlation between IAS and IATS scores (Figure 1, *r* = −0.03, *p* = .48).

Additionally, IATS scores were positively related to the Emotional Awareness and Body Listening scales of the MAIA-2, and negatively related to Not Worrying and Trusting subscales. In turn, IAS scores showed a positive association with the Noticing, Not Distracting, Attention Regulation, Emotional Awareness, Self Regulation, Body Listening and Trusting scales of the MAIA-2. IATS scores were also positively related to the body awareness supradiaphragmatic and subdiaphragmatic scales of the BPQ-SF. A positive association between IAS and the body awareness scale of the BPQ-SF was found, while the association between IAS and the supra- and subdiaphragmatic scales of the BPQ-SF were significantly negative (see Table 3). Regarding the ICQ, IATS scores were positively related, while IAS scores showed a negative relationship.

In relation to self-report measures of subclinical psychopathology, IATS scores were positively related to alexithymia (i.e., TAS-20 scores) and anxiety symptomatology (as indexed by the STAI-T and ASI-3). This relationship, was strongest for the Cognitive subscale of the ASI (*r* = .37, *p* = .003; Somatic: *r* = .25, *p* = .054; Social: *r* = .28, *p* = 0.028). However, no significant relationship between IATS and depressive symptomatology (BDI-II) was found. In contrast, IAS scores were negatively related to TAS-20, STAI-T, and BDI-II scores, but no association was found with ASI-3 scores or its subscales (Cognitive: *r* = −0.17, *p* = .19; Somatic: *r* = −0.82, *p* = .533; Social: *r* = .09, *p* = .48).

When comparing correlations of IAS and IATS scores with other measures of interoception and psychopathology, we observed a stronger association between the body awareness scales of the BPQ-SF and IAS than with IATS scores (Table 4). Furthermore, differences between IAS and IATS and the supra- and subdiaphragmatic scales of the BPQ-SF were also observed. The relations between subclinical psychopathology questionnaires and the IATS scores were generally more positive in comparison with the IAS scores.

### Relation to behavioral measures of interoception

For correlations between IATS, HCT (Samples 2 and 3), and HDT (Sample 2) see Table 5. As visible, the IATS was related neither to IAcc, interoceptive sensibility nor to interoceptive awareness.

## Discussion

The current study aimed at (1) validating the German Version of the IATS and (2) examining the differential relation between subjective interoceptive attention and accuracy scores and other measures of interoception, as well as clinical psychopathology. We demonstrated that the German version of the IATS is a good self-report measure of interoceptive attention. Furthermore, we found that self-reported interoceptive attention and accuracy are not related to each other, and differed in their relation to subclinical measures of psychopathology.

In our samples, the IATS demonstrated a one-factor solution. Although our results may contrast with the original validation of the IATS, in which principal component analysis revealed 3 underlying components, the IATS was initially conceptualized as a one-factor questionnaire. Indeed, scores based on the 3-factor solution seemed to provide inconclusive results (cf. Gabriele et al., 2022). However, we acknowledge that in the current study, some items fit better than others—while Item 13 (i.e., passing wind) had a good fit across all samples, Item 1 (i.e., heartbeat) performed poorly. This indicates that there may be room for improvement and that the questionnaire might benefit from some refining and shortening.

The German version of the IATS demonstrated good internal consistency across versions and samples, however, the measure showed poor test-retest reliability, which contrasts with the results from the original study. Different factors may have influenced the disparity across studies. One aspect is the time elapsed between measurements. Gabriele et al. (2022) examined the test-retest reliability over a period of 30 days. In our sample, the time passed between the test and retest was limited to 200 days and varied across participants, however, subsequent analysis showed that time had little impact on the test-retest reliability. Another important difference between studies is that the current experiment was conducted during the COVID-19 pandemic, which may have notably influenced the stability with which individuals focus on interoceptive signals (Elliott & Pfeifer, 2022). For instance, whether more attention is paid to bodily changes (i.e., in search of possible COVID-symptomatology) may strongly vary from time to time depending on individual exposure to the virus. Future studies considering external factors that could acutely modulate the attentional resources devoted to interoceptive signals may provide more evidence on the stability of subjective interoceptive attention. Importantly, it is still unclear whether self-reported interoceptive attention is a fluctuating state or a stable trait variable.

Although the IATS was specifically designed to match the IAS with regard to interoceptive sensations assessed, in line with the original version of the IATS, we did not find that self-reported interoceptive attention and accuracy were related (Gabriele et al. 2022). Further, we observed a significant relation between IATS scores and the body awareness scale of the BPQ-SF, suggesting that both questionnaires may tap into the same interoceptive construct. However, unlike Gabriele et al. (2022), we observed a positive relation between the body awareness scale of the BPQ-SF and the IAS. Comparative analysis of the relation between questionnaires indicated that scores of the BPQ-SF body awareness scale were more strongly related to IAS than IATS scores. Considering recent findings showing a lack of consistency between different measures of interoception (Desmedt et al., 2022), the low replicability between the BPQ-SF subscale and other self-report measures of interoception across studies may not be surprising. Thus, our findings suggest that the body awareness scale of the BPQ-SF taps more strongly into the subjective accuracy than on the subjective attention construct of interoception.

One of the potential reasons for the divergent relations between the body awareness subscale of the BPQ-SF and measures of interoceptive attention and accuracy across studies may be the subjective interpretation of the scale. Gabriele et al. (2022) observed that interpreting the body awareness scale of the BPQ-SF as a measure of attention or accuracy modulated the association with IATS and IAS scores. Those participants who interpreted the scale as a measure of attention, showed a stronger relationship with IATS than those who interpreted the scale as assessing accuracy, whereas the opposite was true for the relationship with the IAS (Gabriele et al., 2022). Altogether, the lack of consistency in the relation between the body awareness scale of the BPQ-SF and self-report measures of interoceptive attention and accuracy further emphasizes the need for measures assessing different constructs of interoception more precisely (Desmedt et al., 2022).

We did not find evidence for a relationship between the IATS and behavioral measures of interoceptive accuracy indicating that self-reported interoceptive attention is unrelated to the ability to detect one’s own heartbeat. One reason might be that the IATS is targeting a range of interoceptive modalities that might be unrelated to heartbeat detection, such as hunger or pain. However, in other areas, self-report and behavioral tasks often do not show a strong correlation (Cyders & Coskunpinar, 2011). Thus, the findings reported here might also be in line with the notion that self-report and behavioral tasks in the interoceptive domain might tap into different dimensions of interoceptive processing, such as proposed by Murphy et al. (2020). Importantly, we also did not find a relationship between the IAS and behavioral measures of interoception, which further supports this notion.

Regarding measures of subclinical psychopathology, we observed a positive relation between IATS scores and alexithymia and anxious symptomatology. Most importantly, subjective interoceptive accuracy showed a more reduced and/or negative relation to these measures. Extending previous findings (Murphy et al., 2020; Gabriele et al., 2022; Trevisan et al., 2021), our results indicate that subjective interoceptive attention and accuracy contribute differently (sometimes opposingly) to subclinical psychopathology (see also the predictive processing framework; Edwards et al., 2012; Van den Bergh et al., 2017).

The positive association between subjective interoceptive attention and subclinical pathology suggests that the IATS could function as a quick ambulatory assessment of interoception. This fits very well with the growing notion that interoception may be a general underlying factor of psychopathology (Khalsa et al., 2018; Murphy et al., 2019; Quadt et al., 2018). In the same vein, our findings suggest that targeting and reducing dysfunctional interoceptive attention may be a promising way to prevent and/or reduce psychopathology symptomatology. In this sense, Van den Bergh et al. (2021) point out that to achieve more efficient interventions it is important to have an “attentive, open, and non-defensive way of processing threat-relevant information” (Van den Bergh et al., 2021, p. 237). Furthermore, interoceptive exposure therapy has shown promising results in panic disorder (Craske et al., 1997). By non-judgmentally directing (interoceptive) attention toward a threatening stimulus or a fear-inducing situation, the situation can be reevaluated, beliefs updated, and conflicts minimized. Another avenue for interoceptive attention interventions may be mindfulness- and yoga-based interventions, which include interoceptive training elements (Farb et al., 2015; Garland et al., 2012; Pagnoni & Porro, 2014).

Note that our samples consisted mainly of “healthy” young individuals, which might limit the generalization of our findings to clinical samples. Thus, future studies investigating the role of the constructs of interoception in patients suffering from these disorders might provide further insights into the relationship between interoception and psychopathology.

In summary, our results indicate that the IATS is a good instrument for assessing subjective interoceptive attention in the German population. Our findings further provide empirical support for the 2 × 2 factorial model of interoception and emphasize the need to distinguish between different constructs of interoception in relation to subclinical psychopathology and disordered body perception.

## Supplementary Material

Supplementary material Supplemental data for this article can be accessed online at https://doi.org/10.1080/00223891.2024.2316236.

## Figures and Tables

**Table 1:** Overview of samples and demographic information.

Sample	*N* Final participants	*M* age (*SD*)	*N* Female/Male/non-binary
Sample 1	388	30.96 (10.86)	216/166/6
Sample 2	77	23.47 (6.45)	56/20/1
Sample 3	254	24.53 (6.42)	206/48/0

**Table 2:** Summary of the indices of model fit of the one-factor solution for each of the samples.

	X^2^	*df*	*p*	RMSEA	CFI	TLI	SRMR
Sample 3	205.087	185	0.148	0.021	0.995	0.994	0.065
Sample 1 & 3	401.011	185	<.001	0.043	0.980	0.978	0.060

**Table 3:** Correlational analysis between IATS and questionnaire measures and number of participants for each correlation.

Variable	IATS	IAS	MAIA2 noticing	MAIA2 notdis	MAIA2 notwor	MAIA2 attreg	MAIA2 emoaw	MAIA2 selfreg	MAIA2 bodlis	MAIA2 trust	BPQ body awa	BPQ sub	BPQ supra	ICQ	TAS20 Total	ASI Total	STAI	BDI
1.IATS	—																	
	—																	
	642	—																
2. IAS	−0.038	—																
	448	448	—															
3. MAIA2 noticing	0.043	0.457***	—															
	448	448	614	—														
	4. MAIA2 notdis	−0.057	0.099^+^ *	0.014	—														
448		448	614	614	—													
5. MAIA2 notwor		−0.315***	0.032	0.082^+^ *	−0.034	—													
	448	448	614	614	614	—												
	6. MAIA2 attreg	−0.062	0.296***	0.493***	0.101^+^ *	0.236***	—												
448		448	614	614	614	614	—											
7. MAIA2 emoaw		0.170***	0.344***	0.400***	0.084^+^ *	−0.055	0.301***	—											
																	
	448	448	614	614	614	614	614	—										
8. MAIA2 selfreg	0.033	0.172***	0.335***	0.160***	0.177***	0.589***	0.392***	—										
																	
	448	448	614	614	614	614	614	614	—									
9. MAIA2 bodlis	0.093^+^ *	0.237***	0.518***	0.116**	0.043	0.452***	0.424***	0.536***	—									
																	
	448	448	614	614	614	614	614	614	614	—								
10. MAIA2 trust	−0.093^+^ *	0.295***	0.344***	0.135***	0.241***	0.495***	0.223***	0.555***	0.457***	—								
																	
	642	642	614	614	614	614	614	614	614	614	—							
11. BPQ body awa	0.207***	0.317***	0.275***	0.059	−0.049	0.157***	0.268***	0.176***	0.139***	0.079	—							
																	
	642	642	614	614	614	614	614	614	614	614	808	—						
12. BPQ sub	0.336***	−0.125**	−0.017	−0.003	−0.220***	−0.209***	0.040	−0.119**	−0.017	−0.263***	0.171***	—						
	642	642	614	614	614	614	614	614	614	614	808	808	—					
13. BPQ supra	0.337***	−0.081^+^ *	0.067	−0.049	−0.158***	−0.137***	0.094^+^ *	−0.047	0.039	−0.211***	0.216***	0.544***	—					
																	
	448	448	614	614	614	614	614	614	614	614	614	614	614	—				
	0.178***	−0.499***	−0.272***	−0.271***	−0.061	−0.333***	−0.232***	−0.220***	−0.170***	−0.331***	−0.163***	0.326***	0.227***	—				
14. TAS20 Total	60	60	226	226	226	226	226	226	226	226	226	226	226	226	—			
																	
	0.228	−0.454***	−0.310***	−0.366***	−0.295***	−0.422***	−0.297***	−0.378***	−0.334***	−0.442***	−0.177^+^**	0.310***	0.200**	0.562***	—			
15. ASI Total	60	60	226	226	226	226	226	226	226	226	226	226	226	226	226	—		
	0.365**	−0.059	−0.143^+^ *	−0.119	−0.589***	−0.354***	−0.045	−0.334***	−0.089	−0.425***	0.056	0.383***	0.278***	0.311***	0.402***	—		
16. STAI	60	60	226	226	226	226	226	226	226	226	226	226	226	226	226	226	—	
	0.265^+^ *	−0.299^+^ *	−0.210**	−0.138^+^ *	−0.462***	−0.401***	−0.068	−0.508***	−0.274***	−0.631***	−0.069	0.363***	0.245***	0.443***	0.528***	0.569***	—	
17. BDI	60	60	226	226	226	226	226	226	226	226	226	226	226	226	226	226	226	—
	0.146	−0.296^+^ *	−0.263***	−0.134^+^ *	−0.378***	−0.387***	−0.069	−0.426***	−0.233***	−0.592***	−0.058	0.378***	0.274***	0.397***	0.499***	0.464***	0.786***	—

*Note*. Correlations of both versions of the IATS along with the sample size are reported. IATS = Interoceptive Attention Scale; IAS = Interoceptive Accuracy Scale; MAIA2 = Multidimensional Assessment of Interoceptive Awareness Version 2 (Subscales: Noticing; Non-Distracting; Not-Worrying; Attention Regulation; Emotional Awareness; Self-Regulation; Body-Listening; Trusting); BPQ-SF = Body Perception Questionnaire (short form), Body Awa: Body awareness, Supra: Supradiaphragmatic, Sub: Supradiaphragmatic; ICQ = Body Confusion Questionnaire. TAS-20 =Toronto Alexithymia Scale; ASI-3 = Anxiety Sensitivity Inventory; STAI-T = State Trait Anxiety Inventory; BDI-II = Beck’s Depression Inventory.

* *p* < .05,

** *p* < .01,

*** *p* < .001.

+ did not survive Bonferroni correction (*p*<.005).

**Table 4:** Comparison of the relation between IATS and IAS scores with other self-report measures of interoception and subclinical psychopathology.

Self-report measure	Z scores
MAIA2 Noticing	−6.37***
MAIA2 Not Distracting	−2.05*
MAIA2 Not Worrying	−5.10***
MAIA2 Attention Regulation	−2.62**
MAIA2 Emotional Awareness	−5.23***
MAIA2 Self-regulation	−2.07*
MAIA2 Body Listening	−2.15*
MAIA2 Trusting	−5.67***
BPQ-SF Body Awareness	−2.1*
BPQ-SF Supradiaphragmatic	9.02***
BPQ-SF Subdiaphragmatic	8.17***
ICQ	10.39***
TAS-20 Total	8.42***
STAI-T	3.35***
BDI-II	2.53*
ASI-3	2.37*

*Note*. Positive values indicate higher correlations for IATS than IAS. MAIA2 = Multidimensional Assessment of Interoceptive Awareness Version 2, BPQ-SF = Body Perception Questionnaire (short form); ICQ = Interoceptive Confusion Questionnaire, TAS-20=Toronto Alexithymia Scale; STAI-T = State Trait Anxiety Inventory; BDI-II = Beck’s Depression Inventory; ASI-3 = Anxiety Sensitivity Inventory.

* indicates *p* < .05,

** indicates *p* < .01,

*** indicates *p* < .001.

**Table 5:** Correlations between IATS and measures derived from the heartbeat counting task (sample 2 + 3, *N* = 105) and the heartbeat detection task (sample 2).

Variable	*M*	*SD*	1	2	3	4	5
1. IATS	51.36	11.98					
2. IAS	76.50	8.31	.13				
3. HCT accuracy	2.95	1.21	.15	.15			
4. HCT Interoceptive Sensibility	58.20	21.49	−0.04	.21*	−0.21*		
5. HCT Interoceptive Awareness	35.35	25.44	−0.17	.00	−0.70***	.55***	
6. HDT accuracy	−0.39	0.74	−0.03	.07	−0.07	−0.15	−0.06

*Note*. IATS = Interoceptive Attention Scale; IAS = Interoceptive Accuracy Scale; HCT = Heartbeat Counting Task; HDT = Heartbeat Detection task.

* indicates *p* < .05,

** indicates *p* < .01,

*** indicates *p* < .001.
