# Supplementary material for: He Who Seeks Finds (Bodily Signals): German Validation of the Interoceptive Attention Scale (IATS) and its Relationship with Subclinical Psychopathology
Source: J Pers Assess. Author manuscript; Available in PMC 2024 Nov 1. (PMC7616536; doi:10.1080/00223891.2024.2316236)
Supplement: Supplementary material — Supplemental data for this article can be accessed online at https://doi.org/10.1080/00223891.2024.2316236. [file EMS198320-supplement-Supplementary_material.docx]

**Supplementary Information**

1. **German Version of the Interoceptive Attention Scale (IATS)**

Instructions:

*Nachfolgend finden Sie mehrere Aussagen zum Ausmaß der Aufmerksamkeit, die Sie spezifischen körperlichen Empfindungen zukommen lassen. Bitte schätzen Sie auf der untenstehenden Skala ein, wie viel Aufmerksamkeit Sie meinen jeder einzelnen Empfindung zu schenken. Denken Sie dabei daran, wie Sie sich in den meisten Alltagssituationen fühlen, und nicht zu bestimmten Zeitpunkten. Wenn Sie zum Beispiel oft an Ihren Herzschlag, wie hungrig Sie sind, oder ob Sie zur Toilette müssen denken, würden Sie Ihre Aufmerksamkeit für diese Empfindung als hoch einschätzen. Im Gegensatz dazu, wenn Sie nicht oft an Ihren Herzschlag, wie hungrig Sie sind oder ob Sie zur Toilette müssen denken, würden Sie Ihre Aufmerksamkeit für diese Empfindung als niedrig einschätzen.*

*Bitte schätzen Sie nur ein, wie viel Aufmerksamkeit Sie diesen Empfindungen schenken, ungeachtet dessen, wie gut Sie glauben diese wahrzunehmen. Wenn Sie zum Beispiel oft Harndrang verspüren, jedoch auf der Toilette merken, dass Sie gar nicht auf die Toilette müssen, dann würden Sie Ihre Aufmerksamkeit gegenüber diesem Signal trotzdem als hoch einstufen. Konzentrieren Sie sich nicht darauf wie oft Sie denken, dass diese Empfindung tatsächlich in Ihrem Körper stattfindet – wir interessieren uns dafür, wie viel Zeit Sie damit verbringen diesen Empfindungen Aufmerksamkeit zu schenken.*

*Die folgenden Fragen zielen auf die Aufmerksamkeit ab, die Sie Empfindungen schenken, die aus dem Inneren Ihres Körpers kommen. Wenn Ihnen zum Beispiel eine Frage zu Temperatur gestellt wird, bezieht sie sich auf Empfindungen, die Sie innerlich spüren. Es geht nicht darum Ihre Hände zu berühren, um zu spüren wie warm Ihre Haut ist. Wenn Ihnen eine Frage zu Ihrem Herzschlag gestellt wird, bezieht sie sich auf Gefühle, die Sie in Ihrem Körper spüren, ohne Ihren Puls zu messen.*

1. Die meiste Zeit ist meine Aufmerksamkeit darauf fokussiert, ob mein Herz schnell schlägt.
2. Die meiste Zeit ist meine Aufmerksamkeit darauf fokussiert, ob ich hungrig bin.
3. Die meiste Zeit ist meine Aufmerksamkeit darauf fokussiert, ob ich schnell atme.
4. Die meiste Zeit ist meine Aufmerksamkeit darauf fokussiert, ob ich durstig oder dehydriert bin.
5. Die meiste Zeit ist meine Aufmerksamkeit darauf fokussiert, ob ich urinieren muss.
6. Die meiste Zeit ist meine Aufmerksamkeit darauf fokussiert, ob ich koten/defäkieren muss.
7. Die meiste Zeit, wenn ich esse, ist meine Aufmerksamkeit auf verschiedene Geschmäcker fokussiert.
8. Die meisten Zeit ist meine Aufmerksamkeit darauf fokussiert, ob mir schlecht ist oder ich mich übergeben muss.
9. Die meiste Zeit ist meine Aufmerksamkeit darauf fokussiert, ob ich niesen muss.
10. Die meiste Zeit ist meine Aufmerksamkeit darauf fokussiert, ob ich husten muss.
11. Die meiste Zeit ist meine Aufmerksamkeit auf meine Körpertemperatur fokussiert (ob mir heiß oder kalt ist).
12. Die meiste Zeit ist meine Aufmerksamkeit darauf fokussiert, ob ich sexuell erregt bin.
13. Die meiste Zeit ist meine Aufmerksamkeit darauf fokussiert, ob ich pupsen muss.
14. Die meiste Zeit ist meine Aufmerksamkeit darauf fokussiert, ob ich rülpsen muss.
15. Die meiste Zeit ist meine Aufmerksamkeit darauf fokussiert, ob meine Muskeln müde sind oder ich einen Muskelkater habe.
16. Die meiste Zeit ist meine Aufmerksamkeit darauf fokussiert, ob ich Schmerzen habe, nachdem ich mich verletzt habe.
17. Die meiste Zeit ist meine Aufmerksamkeit darauf fokussiert, ob ich Schmerzen habe (nicht durch eine Verletzung verursacht).
18. Die meiste Zeit ist meine Aufmerksamkeit darauf fokussiert, ob mein Blutzucker niedrig ist.
19. Die meiste Zeit, wenn mich jemand berührt, ist meine Aufmerksamkeit darauf fokussiert, ob es sich angenehm/liebevoll anfühlt.
20. Die meiste Zeit ist meine Aufmerksamkeit darauf fokussiert, ob sich Berührungen oder Materialien kitzelig anfühlen.
21. Die meiste Zeit ist meine Aufmerksamkeit darauf fokussiert, ob mein Körper juckt.

*Scale: Stimme überhaupt nicht zu; Stimme nicht zu; Stimme weder zu noch lehne ab; Stimme zu; Stimme stark zu.*
